# Polysaccharide from *Gleditsia sinensis* Seed Endosperm Ameliorates Type 2 Diabetes and Its Associated Cardiorenal Injuries by Modulating TLR4/MyD88/NF-κB Pathway and Gut Microbiota

**DOI:** 10.3390/metabo16050339

**Published:** 2026-05-18

**Authors:** Mei Liu, Wenping Liao, Hongyun Liu, Feng Xu, Yanyan Zhang, Xiangpei Wang, Hongmei Wu

**Affiliations:** 1 School of Pharmacy, Guizhou University of Traditional Chinese Medicine, Guiyang 550025, China; 2 School of Chinese Ethnic Medicine, Guizhou Minzu University, Guiyang 550025, China

**Keywords:** *Gleditsia sinensis* seed endosperm polysaccharide, type 2 diabetes mellitus, cardiorenal injuries, gut microbiota, TLR4/MyD88/NF-κB pathway, short-chain fatty acids

## Abstract

Background: Type 2 diabetes mellitus (T2DM) represents a pressing global health challenge, underscoring the urgency of developing effective dietary interventions derived from natural resources. Zaojiaomi polysaccharide (ZJMP) from the endosperm of *Gleditsia sinensis* seeds (zaojiaomi), a traditional edible product, exhibits largely underexplored potential in T2DM management. Methods: In the present study, the antidiabetic effects and underlying mechanisms of ZJMP were investigated using a rat model of T2DM induced by a high-fat diet (HFD) combined with streptozotocin (STZ). Relevant biochemical indicators were detected, and histopathological examination was performed. The expression levels of key components of the TLR4/MyD88/NF-κB signaling pathway, as well as the inflammatory cytokines IL-6 and IL-1β in renal tissues, were further analyzed. Additionally, gut microbiota composition and the levels of short-chain fatty acids were determined. Results: ZJMP treatment significantly ameliorated hyperglycemia and dyslipidemia, elevated serum insulin levels, reduced intestinal mucosal permeability, and attenuated histopathological lesions in the heart, kidney, and pancreas of T2DM rats. Meanwhile, ZJMP notably alleviated renal inflammation by suppressing the production of IL-1β and IL-6, as well as inhibiting the TLR4/MyD88/NF-κB pathway. Furthermore, ZJMP administration effectively modulated gut microbiota composition and increased fecal concentrations of acetic acid and propionic acid. Conclusions: Collectively, these findings elucidate the novel bioactivity of ZJMP and highlight its potential as a promising functional food ingredient or dietary supplement for T2DM management.

## 1. Introduction

Type 2 diabetes mellitus (T2DM) is a progressive metabolic disorder and an escalating global public health crisis. Its age-standardized prevalence rises annually, with projections indicating that over 1.27 billion people worldwide will be affected by 2050 [1]. T2DM is characterized by low-grade inflammation, persistent hyperglycemia, and insulin resistance [2]. Its pathogenesis is complex, which poses a serious threat to patients’ quality of life. Additionally, T2DM constitutes a significant risk factor for several irreversible comorbidities, including nephropathy, cardiomyopathy, and retinopathy [3]. Currently, clinically used oral antidiabetic medications include glucosidase inhibitors, thiazolidinediones, and biguanides [4]. These drugs, however, are expensive and may trigger adverse reactions including gastrointestinal disturbances, hypoglycemia, and weight gain [5]. They not only cause physical and psychological distress to patients but also impose a heavy economic burden. Consequently, the identification of low-toxic, cost-effective, and efficacious therapeutic agents is urgently required to delay disease progression.

Modern research has demonstrated that galactomannans, primarily extracted from the endosperms of leguminous seeds, exhibit considerable application potential in the food industry [6]. As a traditional food ingredient, zaojiaomi (the endosperm of *Gleditsia sinensis* Lam. seeds) has garnered growing attention owing to its high content of bioactive polysaccharides (zaojiaomi polysaccharides, ZJMP). ZJMP is a neutral galactomannan with a backbone of β-(1→4)-linked mannose residues and side chains of α-(1→6)-linked galactose residues, with its major monosaccharide constituents being mannose and galactose [7,8,9]. To date, most investigations into galactomannans derived from leguminous plants have focused on their direct hypoglycemic effects [10,11,12] and antioxidant activity [13]. Furthermore, a recent study has reported that galactomannans isolated from *Gleditsia* species seeds possess prominent hypoglycemic, hypolipidemic, and anti-inflammatory properties [14]. However, the underlying mechanisms by which ZJMP alleviates T2DM requires deeper elucidation, and the effects of ZJMP on cardiac and renal damage induced by T2DM remain to be elucidated.

Gut microbiota dysbiosis and chronic inflammation are closely correlated with T2DM and its associated complications [15,16]. Targeted modulation of the gut microbiota can alleviate the pathogenic processes underlying T2DM and its related complications [17,18,19]. Notably, the Toll-like receptor 4/myeloid differentiation factor 88/nuclear factor-κB (TLR4/MyD88/NF-κB) pathway acts as a pivotal regulator of inflammatory cascades linked to T2DM progression. Emerging evidence suggests that selective inhibition of this pathway can mitigate diabetic nephropathy, a prevalent and debilitating complication of T2DM [20]. Growing evidence indicates that numerous natural polysaccharides exert beneficial effects on managing T2DM and its related complications [21,22,23]. Collectively, these findings support the potential of a dual-targeted strategy that modulates both gut microbiota and inflammatory responses to slow T2DM progression and alleviate its associated complications.

It is noteworthy that the tumor necrosis factor-α (TNF-α) signaling pathway also plays an essential regulatory role in the inflammatory pathological progression of T2DM and its complications. Nevertheless, TNF-α serves as one of the downstream effector molecules following activation of the canonical TLR4/MyD88/NF-κB inflammatory pathway. Aberrant activation of this signaling pathway acts as the pivotal initiating link that drives the massive release of multiple pro-inflammatory cytokines including TNF-α. Therefore, this study mainly focuses on the upstream core inflammatory regulatory pathway TLR4/MyD88/NF-κB to elucidate the mechanism underlying the protective effect of ZJMP, rather than performing an independent in-depth exploration of the TNF-α pathway.

Given the above rationale, we hypothesized that ZJMP alleviates T2DM and its associated cardiorenal injuries by modulating gut microbiota dysbiosis and suppressing the TLR4/MyD88/NF-κB pathway. Distinct from previous studies that mainly focused on the hypoglycemic and antioxidant capacities of legume-derived galactomannans, this study is the first to simultaneously explore the underlying mechanisms by focusing on the TLR4/MyD88/NF-κB pathway and the gut microbiota. Additionally, the effects of ZJMP on short-chain fatty acids (SCFAs) were analyzed. Collectively, this work provides experimental evidence and a theoretical basis for the development of ZJMP as a natural functional food ingredient for the management of T2DM and T2DM-induced cardiorenal injuries.

## 2. Materials and Methods

### 2.1. Drugs and Reagents

Streptozotocin (STZ) was obtained from Sigma-Aldrich Co., LLC. (St. Louis, MO, USA). Metformin hydrochloride tablets (Approval No.: H20023370) were purchased from Sino-US Shanghai Squibb Pharmaceutical Co., Ltd. (Shanghai, China). D-mannose, D-xylose, D-galactose, and D-glucose were purchased from Push Bio-Technology Co., Ltd. (Chengdu, China). Standard reference materials of acetic acid, propionic acid, and butyric acid were purchased from Chroma Biotechnology Co., Ltd. (Chengdu, China). Antibodies against TLR4, MyD88 (Abcam, UK), β-actin (Proteintech Group, Inc., Wuhan, China) and NF-κB p65 (Cell Signaling Technology, Danvers, MA, USA) were acquired for the experiment.

### 2.2. Extraction of ZJMP

Zaojiaomi was purchased from Zhijin County, Bijie City, Guizhou Province, and identified by Professor Xiangpei Wang of Guizhou Minzu University as the processed and dried endosperm obtained by removing the seed coats and cotyledons from the dried mature seeds of *Gleditsia sinensis* Lam. Zaojiaomi was first crushed, and the resulting powder was mixed with neutral protease at a mass ratio of 4:1. The mixture was soaked in water for 24 h, followed by heating under reflux at 80 °C for 2 h. The extraction was performed only once. After suction filtration and concentration of the filtrate, anhydrous ethanol was added at a volume ratio of 1:4. The mixture was statically placed at 4 °C overnight to induce flocculent precipitation, which was then collected by centrifugation and filtration. The ethanol solution was discarded, and the obtained precipitate was washed with anhydrous ethanol and dried by evaporation to yield ZJMP. It should be noted that no dedicated deproteinization step was included, and no further purification was carried out other than ethanol washing. The extraction yield of ZJMP was 40.31%.

### 2.3. Analytical Methods for ZJMP Characterization

The molecular weight and homogeneity of each fraction were analyzed using size exclusion chromatography coupled with multi-angle light scattering and refractive index detection (SEC-MALS-RI). Prior to sample analysis, the SEC-MALS-RI system was fully validated using standard pullulan to ensure the stability and accuracy of the system throughout the entire measurement process. The system (DAWN HELEOS-II, Wyatt Technology, Santa Barbara, CA, USA; Optilab T-rEX, Wyatt Technology, Santa Barbara, CA, USA) was operated with a mobile phase of 0.1 M NaNO_3_ aqueous solution containing 0.02% NaN_3_ at a flow rate of 0.6 mL/min. Separation was performed on two tandem Shodex OH-pak columns (SB-805 and SB-803, 300 × 8 mm; Showa Denko, Tokyo, Japan) maintained at 45 °C. A dn/dc value of 0.141 mL/g was used to calculate the weight-average molecular weight (Mw), number-average molecular weight (Mn), and polydispersity index (Mw/Mn). Of note, molecular weight measurement was performed once.

The monosaccharide composition of ZJMP was measured by high-performance liquid chromatography (HPLC). Briefly, 0.301 g of ZJMP was dissolved in 3 mL of 72% sulfuric acid and incubated at 30 °C for 1 h. Subsequently, 85 mL of water was added to the mixture, which was autoclaved at 121 °C for 60 min to hydrolyze into monosaccharides. The hydrolyzate was neutralized with calcium carbonate, centrifuged, and the supernatant was mixed sequentially with sodium hydroxide solution and 1-phenyl-3-methyl-5-pyrazolone (PMP)-methanol solution. After heating in a 70 °C water bath for 1 h, the mixture was cooled, treated with hydrochloric acid solution followed by chloroform, vortexed, extracted twice, and centrifuged (12,000 rpm, 1 min). The upper aqueous phase was collected and filtered through a 0.22 μm microporous membrane for subsequent HPLC analysis, with detailed chromatographic parameters provided in the Supplementary Materials.

The structure of ZJMP was analyzed using Fourier transform infrared (FT-IR) spectroscopy. Briefly, 1 mg of ZJMP was thoroughly ground with 100 mg of dried KBr. The mixture was then compressed into a transparent pellet under a pressure of 0.6 T for 1 min using a pellet press. The pellet was immediately scanned using an FT-IR spectrometer.

### 2.4. Animals and Experimental Design

Thirty-six 6-week-old male Sprague-Dawley rats (180–220 g) were purchased from Changsha Tianqin Biotechnology Co., Ltd. (Changsha, China). The rats were housed under SPF conditions (12 h light/dark cycle, 55–70% relative humidity, 23 ± 2 °C) with free access to a standard diet and drinking water. Six SD rats were randomly allocated to the Control group and fed a standard diet, while all remaining rats were administered a high-fat diet for 4 weeks prior to T2DM induction via two consecutive intraperitoneal injections of STZ at 35 mg/kg and 30 mg/kg on successive days. Rats in the Control group were treated with an equivalent volume of buffer solution. The T2DM model was successfully established one week after the second STZ injection, as evidenced by a fasting blood glucose (FBG) level of ≥11.1 mmol/L in tail vein blood samples.

The successfully established T2DM rat models were randomly assigned to five subgroups: T2DM (model), Met (metformin hydrochloride), DTH (high-dose ZJMP), DTM (medium-dose ZJMP), and DTL (low-dose ZJMP), with six rats per group. For 4 consecutive weeks, the Met group was gavaged with metformin hydrochloride at 200 mg/kg, and the DTH, DTM, and DTL groups received ZJMP at 472, 236, and 118 mg/kg, respectively. FBG levels and general health status of the rats were monitored at regular intervals. All experimental protocols were approved by the Animal Ethics Committee of Guizhou University of Traditional Chinese Medicine (Approval No. 20220013).

### 2.5. Sample Collection and Serum Biochemical Analysis

Four weeks after administration, the rats were anesthetized via isoflurane inhalation, with blood collected from the abdominal aorta and centrifuged at 3500 rpm for 15 min after 2 h of room temperature incubation. Serum levels of diamine oxidase (DAO), insulin (INS), and D-lactic acid (D-LA) were quantified according to the instructions provided by Elabscience Biotechnology Co., Ltd. (Wuhan, China). Glycated hemoglobin A1c (HbA1c), total cholesterol (TC), low-density lipoprotein cholesterol (LDL-C), triglyceride (TG), creatinine (Cr), uric acid (UA), and high-density lipoprotein cholesterol (HDL-C) were determined using commercial kits from Nanjing Jiancheng Bioengineering Institute (Nanjing, China), following the manufacturer’s protocols. Lactate dehydrogenase (LDH) and creatine kinase MB isoenzyme (CK-MB) activities were measured in accordance with guidelines from Mindray Biomedical Electronics Co., Ltd. (Shenzhen, China).

### 2.6. Determination of Proinflammatory Cytokines

Interleukin-1β (IL-1β) and interleukin-6 (IL-6) levels in renal tissue homogenates were determined using respective ELISA kits (Neobioscience Technology Company, Shenzhen, China), strictly adhering to the manufacturers’ recommended procedures.

### 2.7. Histopathological Analysis

For histological analysis, the pancreatic, cardiac, and renal tissues were fixed in 4% paraformaldehyde, followed by dehydration, paraffin embedding, and sectioning into slices. The tissue architecture and pathological changes were subsequently observed and evaluated in hematoxylin and eosin (H&E)-stained sections under a light microscope.

### 2.8. Western Blotting

Rat kidney tissues from each group were lysed for total protein extraction, and the protein concentration was subsequently determined using a BCA protein quantification kit (Biosharp, Hefei, China). The supernatants of the samples were subjected to protein denaturation, separated by SDS-PAGE, and electrotransferred onto PVDF membranes. After blocking, the membranes were incubated with the primary antibodies at 4 °C followed by the secondary antibodies at 25 °C. The immunoreactive bands were visualized via chemiluminescence development and exposure, with images captured using a gel imaging system. The gray values of the bands were quantified using ImageJ software (version 1.51j), and the results were expressed as the relative expression levels of the target proteins.

### 2.9. Gut Microbiota Analysis

Total DNA was extracted from rat fecal samples using the CTAB/SDS method, and its concentration and purity were assessed by agarose gel electrophoresis. The extracted DNA was used as the template for PCR amplification. The resulting PCR products were subjected to agarose gel electrophoresis, then recovered and purified using the Qiagen Gel Extraction Kit (Qiagen, Hilden, Germany). Subsequently, a sequencing library was constructed and its quality verified. Finally, high-throughput sequencing was performed on the Illumina NovaSeq platform.

### 2.10. Determination of SCFAs by HPLC

An amount of 0.5 g of rat fecal samples was homogenized in 2 mL of purified water, followed by vortexing and centrifugation at 12,000× *g* for 20 min. The resulting supernatant was acidified with 200 μL of hydrochloric acid and statically extracted with 7 mL of diethyl ether for 20 min, followed by centrifugation at 3000× *g* for 10 min; the upper organic phase was mixed with 1 mL of NaOH solution and re-extracted and centrifuged under the same conditions for 20 min and 10 min, respectively. The lower aqueous layer was collected, re-acidified with 200 μL hydrochloric acid, mixed uniformly, adjusted to a final volume of 1.5 mL with purified water, and subsequently passed through a 0.22 μm microporous membrane. Detailed chromatographic parameters are provided in the Supplementary Materials.

### 2.11. Statistical Analysis

Data are presented as the mean ± standard deviation (SD) and analyzed via SPSS 26.0. Prior to one-way ANOVA, all data were subjected to a normality test using the Shapiro–Wilk test and homogeneity of variance test using Levene’s test. When data met the assumptions of normality and homogeneity of variance, one-way ANOVA was used for multi-group comparisons, followed by the LSD test for post hoc pairwise comparisons. Tamhane’s T2 test was applied for data with heterogeneous variance. The LSD test was selected in this study due to its good statistical sensitivity and suitability for pairwise comparison among groups when variance homogeneity is confirmed via Levene’s test. Considering that multiple biomarkers were detected and analyzed simultaneously, each biomarker was statistically evaluated independently within its own one-way ANOVA framework; therefore, no additional multiple comparison correction was performed across different biomarkers. *p* < 0.05 was considered statistically significant. Data visualization was performed with GraphPad Prism 8.0.

## 3. Results

This study systematically performed the structural characterization of ZJMP, the detection of related biochemical indicators, histopathological observation, and the expression analysis of the TLR4/MyD88/NF-κB signaling pathway, as well as the inflammatory cytokines IL-6 and IL-1β in renal tissues. Additionally, from the perspective of intestinal microecology, this study explored changes in gut microbiota composition and SCFA levels. These results suggest that ZJMP alleviates T2DM and related cardiorenal injuries by regulating biochemical indexes, modulating gut microbiota structure, promoting SCFA production, and inhibiting the activation of the TLR4/MyD88/NF-κB inflammatory pathway. Since the core molecules of the TLR4/MyD88/NF-κB pathway (including TLR4, MyD88 and NF-κB) and its downstream pro-inflammatory cytokines (IL-6 and IL-1β) were already examined, and the TNF-α pathway shares partial molecular overlap and signaling crosstalk with the above pathway, relevant indicators of the TNF-α pathway were not independently evaluated in the present study. Nevertheless, given the crucial role of TNF-α signaling in diabetic pathological progression, its potential function in the anti-inflammatory action of ZJMP deserves in-depth exploration in future research.

### 3.1. Characterizations of ZJMP

**Figure 1:**
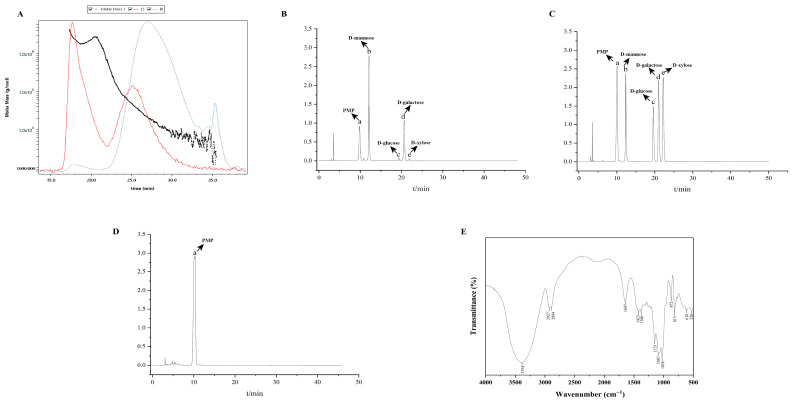
Characterizations of ZJMP. (**A**) Molecular weights of ZJMP; (**B**) HPLC chromatograms of ZJMP; (**C**) HPLC chromatograms of monosaccharide standards; (**D**) FT-IR spectrum of ZJMP. ZJMP: zaojiaomi polysaccharide; HPLC: high-performance liquid chromatography; (**E**) FT-IR: Fourier transform infrared spectroscopy.

The molecular weight characteristics of ZJMP were analyzed by gel permeation chromatography. The Mw and Mn were determined to be 59.92 kDa and 14.73 kDa, respectively, with a Mw/Mn of 4.07 (Figure 1A).

ZJMP was extracted from Zaojiaomi with a purity of 86.64%. As a primary galactomannan composed mainly of galactose and mannose, its monosaccharide composition is shown in Figure 1B–D. HPLC chromatogram of the fully hydrolyzed ZJMP revealed the presence of D-mannose (58.59%), D-glucose (2.23%), D-galactose (28.05%), and D-xylose (1.51%).

FT-IR analysis was performed to characterize the functional groups of the polysaccharide (Figure 1E). A broad and strong absorption band at 3394 cm^−1^ is attributed to O–H stretching vibrations. The intensive peaks at 1151, 1087, and 1026 cm^−1^ were characteristic of C–O–C glycosidic linkages and C–OH stretching vibrations, indicating the existence of pyranose residues. Significantly, the characteristic absorption bands at 872 cm^−1^ and 813 cm^−1^ were C–H bending vibrations of β-mannopyranose residues, which is consistent with previous research [8].

### 3.2. ZJMP Improves FBG, HbA1c, and INS in T2DM Rats

**Figure 2:**
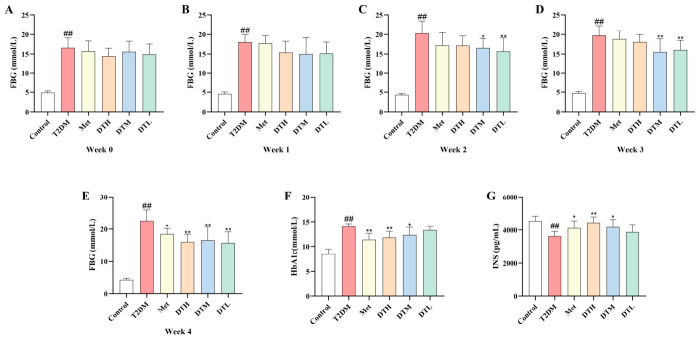
Impacts of ZJMP on FBG, HbA1c, and INS levels in rats with T2DM. (**A**–**E**) FBG; (**F**) HbA1c; (**G**) INS. (*n* = 6). ^##^ *p* < 0.01 vs. Control; * *p* < 0.05, ** *p* < 0.01 vs. T2DM. ZJMP: zaojiaomi polysaccharide; T2DM: type 2 diabetes mellitus; FBG: fasting blood glucose; HbA1c: Glycated hemoglobin A1c; INS: insulin.

The T2DM group showed persistently higher FBG levels than the Control group throughout the 4-week intervention (Figure 2A–E). Notably, all treatment groups showed a significant reduction in FBG after 4 weeks of intervention (Figure 2E). With respect to HbA1c, the T2DM group had a markedly higher level, while interventions with Met, DTH and DTM effectively reduced HbA1c levels (Figure 2F). The significant decrease in INS levels in the T2DM group was effectively reversed by Met, DTH and DTM treatments, whereas the DTL group only showed a non-significant increasing trend (Figure 2G). Collectively, ZJMP intervention exerted a significant hypoglycemic effect and effectively ameliorated hyperglycemia in T2DM rats.

### 3.3. ZJMP Ameliorates Dyslipidemia in T2DM Rats

**Figure 3:**
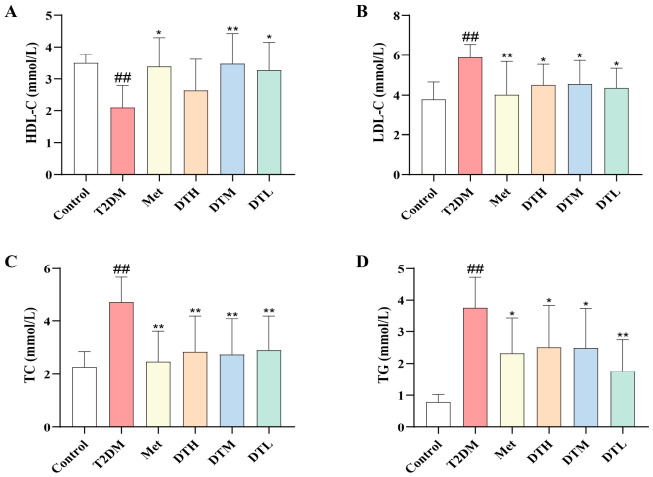
Effects of ZJMP on lipid levels in T2DM rats. (**A**) HDL-C; (**B**) LDL-C; (**C**) TC; (**D**) TG. (*n* = 6). ^##^ *p* < 0.01 vs. Control; * *p* < 0.05, ** *p* < 0.01 vs. T2DM. ZJMP: zaojiaomi polysaccharide; T2DM: type 2 diabetes mellitus; HDL-C: high-density lipoprotein cholesterol; LDL-C: low-density lipoprotein cholesterol; TC: total cholesterol; TG: triglyceride.

Analysis of lipid metabolic parameters revealed a distinct dyslipidemic phenotype in the T2DM group, which was characterized by markedly elevated levels of LDL-C, TC and TG, accompanied by reduced HDL-C levels (Figure 3A–D). Following four weeks of treatment, all treatment groups exhibited a significant reduction in LDL-C, TC and TG levels (Figure 3B–D). Specifically, the Met, DTM and DTL groups showed a notable increase in HDL-C levels, whereas the DTH group showed an upward trend in HDL-C with no statistically significant differences (Figure 3A). These results demonstrate that ZJMP exerts beneficial regulatory effects on dyslipidemia in T2DM rats.

### 3.4. ZJMP Improves Intestinal Mucosal Injury Markers in T2DM Rats

**Figure 4:**
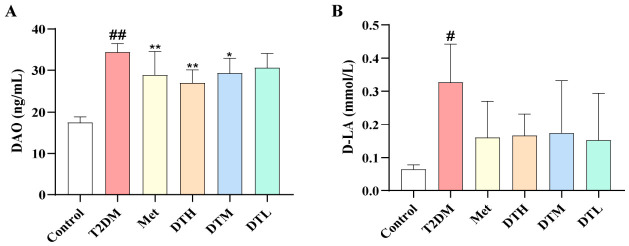
Impacts of ZJMP on intestinal mucosal injury in rats with T2DM. (**A**) DAO; (**B**) D-LA. (*n* = 6). ^#^ *p* < 0.05, ^##^ *p* < 0.01 vs. Control; * *p* < 0.05, ** *p* < 0.01 vs. T2DM. ZJMP: zaojiaomi polysaccharide; T2DM: type 2 diabetes mellitus; DAO: diamine oxidase; D-LA: D-lactic acid.

The T2DM group showed significantly elevated serum DAO and D-LA levels compared with the Control group, indicating enhanced intestinal mucosal permeability in T2DM rats. The intervention resulted in reduced DAO and D-LA levels in all treated groups. Specifically, the Met, DTH, and DTM groups exhibited statistically significant decreases in DAO activity, whereas the DTL group only showed a non-significant downward trend. In contrast, no significant reduction in D-LA levels were observed across all treatment groups (Figure 4A,B). These observations suggest that ZJMP exerted a partial improving effect on intestinal mucosal injury in T2DM rats.

### 3.5. ZJMP Improves Cardiac and Renal Biochemical Markers in T2DM Rats

**Figure 5:**
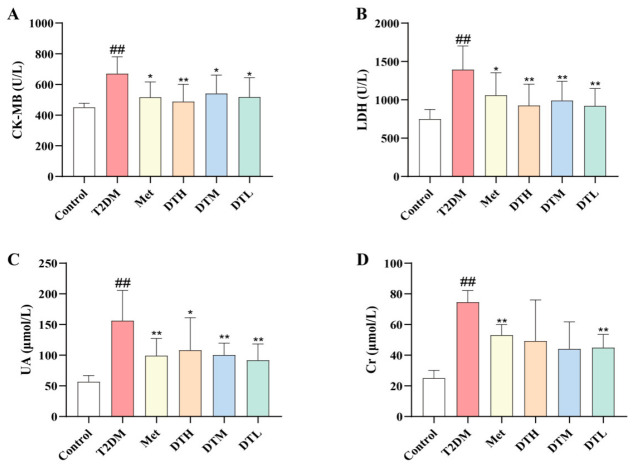
Effects of ZJMP on cardiac and renal injury in T2DM rats. (**A**) CK-MB; (**B**) LDH; (**C**) UA; (**D**) Cr. (*n* = 6). ^##^ *p* < 0.01 vs. Control; * *p* < 0.05, ** *p* < 0.01 vs. T2DM. ZJMP: zaojiaomi polysaccharide; T2DM: type 2 diabetes mellitus; CK-MB: creatine kinase MB isoenzyme; LDH: lactate dehydrogenase; UA: uric acid; Cr: creatinine.

Figure 5A–D demonstrates that the induction of T2DM markedly elevated CK-MB, LDH, and UA levels compared with the Control group, and these abnormal elevations were significantly reversed following intervention with Met, DTH, DTM or DTL. Specifically, serum Cr levels were significantly decreased in the Met and DTL groups, while the DTH and DTM groups showed a non-significant downward trend relative to the T2DM group. These results suggest that ZJMP improves biomarkers related to cardiac and renal injury.

### 3.6. ZJMP Alleviates Diabetes-Associated Histopathological Lesions in the Heart, Kidneys, and Pancreas

**Figure 6:**
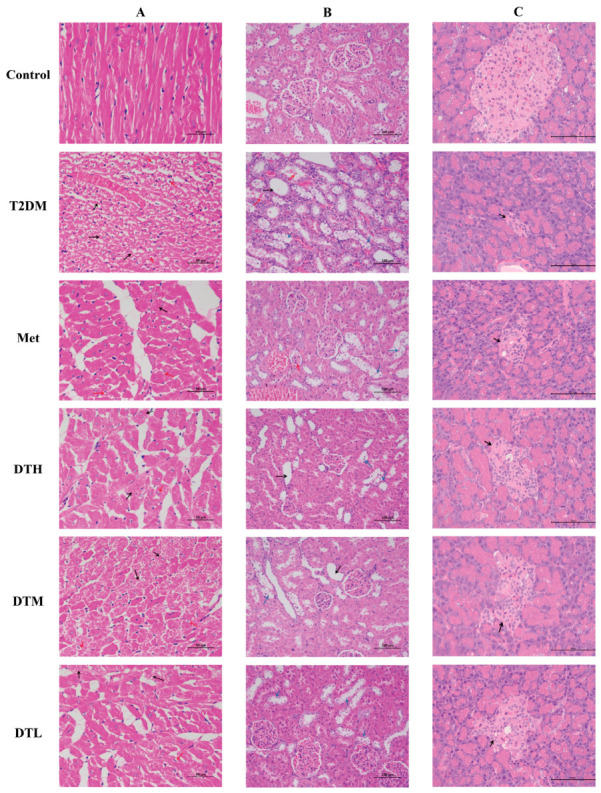
Representative HE staining images. (**A**) Cardiac pathological sections (×400). Black arrows indicate cytoplasmic granular degeneration. Red arrows indicate nuclear loss or perinuclear vacuolar degeneration. (**B**) Renal pathological sections (×200). Black arrows indicate dilatation of the renal tubular lumen. Red arrows indicate cell debris. Blue arrows indicate atrophy, swelling or vacuolation of renal tubular epithelial cells. (**C**) Pancreatic pathological sections (×400). Black arrows indicate the islet area. HE: hematoxylin and eosin.

Histopathological examination revealed varying degrees of pathological damage in the cardiac, renal, and pancreatic tissues of T2DM-induced rats (Figure 6A–C). Cardiac sections from the T2DM group showed myocardial cells with varying degrees of blurred boundaries and ill-defined morphological structures beneath the epicardium and endocardium, accompanied by cytoplasmic granular degeneration, nuclear pyknosis or karyolysis, as well as perinuclear vacuolization. In the T2DM group, renal tissues presented obvious segmental tubular lesions and mild glomerular lesions, characterized by tubular dilatation, atrophy and vacuolar swelling of renal tubular epithelial cells, basophilic degeneration, and segmental glomerular hyalinosis. H&E staining demonstrated a significant reduction in both the size and number of pancreatic islets in T2DM model rats. Additional morphological abnormalities included disorganized arrangement of islet cells, unclear islet boundaries, increased deposition of eosinophilic cytoplasmic granules, and perinuclear vacuolar degeneration in partial islet cells. Interventions with Met, DTH, DTM, and DTL alleviated pathological damage in the heart, kidney, and pancreas to varying extents, thus confirming the protective effect of ZJMP against T2DM-induced histological damage in these organs.

### 3.7. ZJMP Alleviates Renal Inflammation in T2DM Rats

**Figure 7:**
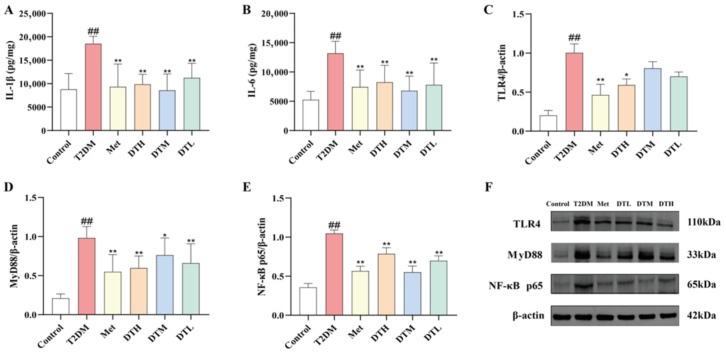
Effects of ZJMP on inflammatory factors and TLR4/MyD88/NF-κB pathway. (**A**) IL-1β; (**B**) IL-6; (**C**) protein expression of TLR4; (**D**) protein expression of MyD88; (**E**) protein expression of NF-κB p65; (**F**) protein expression bands of TLR4, MyD88, and NF-κB p65. (*n* = 6). ^##^ *p* < 0.01 vs. Control; * *p* < 0.05, ** *p* < 0.01 vs. T2DM. ZJMP: zaojiaomi polysaccharide; T2DM: type 2 diabetes mellitus; IL-1β: interleukin-1β; IL-6: interleukin-6; TLR4: toll-like receptor 4; MyD88: myeloid differentiation factor 88; NF-κB p65: nuclear factor-κB p65.

As shown in Figure 7A,B, renal IL-1β and IL-6 levels were significantly elevated in the T2DM group relative to the Control group, and Met, DTL, DTM and DTH interventions markedly reduced the levels of these cytokines. The T2DM group exhibited a significant upregulation of TLR4, MyD88 and NF-κB p65 expression relative to the Control group, an effect effectively reversed by Met treatment. Similarly, DTH, DTM and DTL treatments significantly decreased MyD88 and NF-κB p65 expression; for TLR4, the DTH group showed a significant reduction, while the DTM and DTL groups had a non-significant downward trend (Figure 7C–F).

### 3.8. ZJMP Regulates Gut Microbiota in T2DM Rats

**Figure 8:**
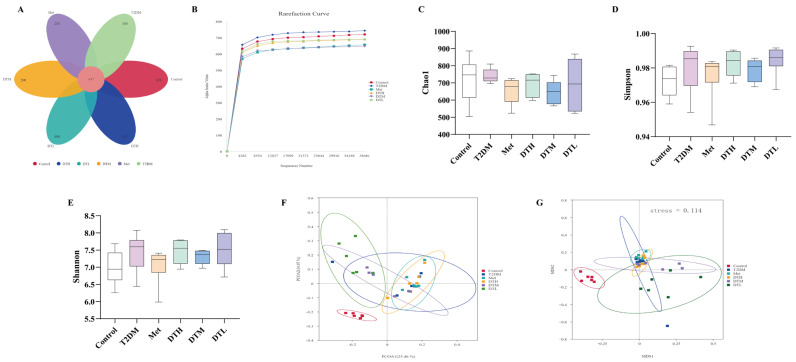
Effects of ZJMP on gut microbiota in T2DM rats. (**A**) Petal plot (the pink section indicates the shared ASVs among groups); (**B**) rarefaction curve; (**C**) Chao index; (**D**) Simpson index; (**E**) Shannon index; (**F**) PCoA plot; (**G**) NMDS plot. (*n* = 6). ZJMP: zaojiaomi polysaccharide; T2DM: type 2 diabetes mellitus; PCoA: principal coordinates analysis; NMDS: non-metric multidimensional scaling.

The petal map (Figure 8A) revealed 437 ASVs shared across six groups, with marked variations in unique ASV numbers: 658 (Control), 389 (T2DM), 255 (Met), 267 (DTH), 290 (DTM), and 696 (DTL). The rarefaction curves showed that with increasing sequencing depth, the curves gradually plateaued, indicating adequate sequencing effort to capture the full species diversity of the samples (Figure 8B). No significant differences in alpha diversity were detected among the T2DM, Control, and all treatment groups (Figure 8C–E).

Subsequently, we systematically compared gut microbial communities across samples via PCoA and NMDS to assess their similarities and differences. PCoA (Figure 8F) revealed that the top two principal components of the fecal microbiota explained 23.46% and 16.07% of the total variance across the six groups, respectively. Samples from each group exhibited obvious visual clustering, indicating that rats within the same group shared similar gut microbial structures. Further analysis revealed an apparent visual separation in gut microbiota composition between the Control and T2DM groups, with the ZJMP group appearing closer to that of the Control group. The NMDS results were consistent with those of PCoA (Figure 8G).

**Figure 9:**
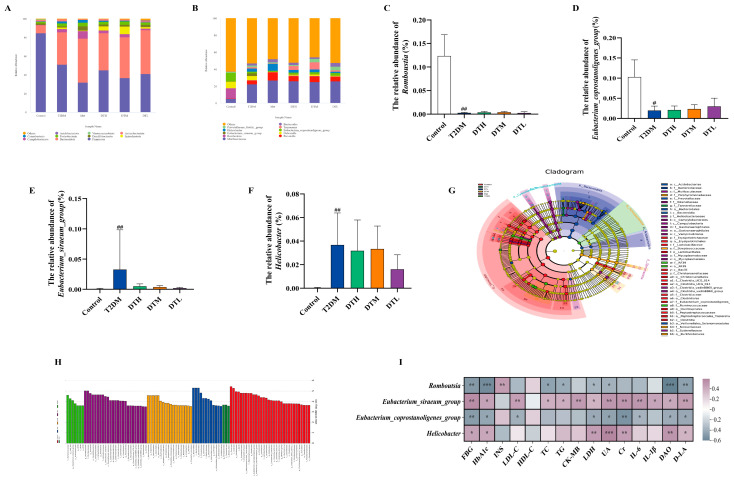
Impacts of ZJMP on gut microbiota relative abundance and associated correlation analysis. (**A**,**B**) relative abundance at the phylum and genus levels. (**C**–**F**) Relative abundance of *Romboutsia*, *Eubacterium coprostanoligenes* group, *Eubacterium siraeum* group, and *Helicobacter*. (**G**) Evolutionary cladogram. (**H**) LDA value distribution histogram among groups. (**I**) Correlation between gut microbiota and T2DM-associated biomarkers. (*n* = 6).^#^ *p* < 0.05, ^##^ *p* < 0.01 vs. Control. * *p* < 0.05, ** *p* < 0.01, *** *p* < 0.001, ZJMP: zaojiaomi polysaccharide; LDA: linear discriminant analysis.

To elucidate how ZJMP modulates the gut microbiota, we analyzed shifts in microbial abundance. Firmicutes, Bacteroidota, Campylobacterota, and Spirochaetota dominated the fecal microbial community across the six experimental groups at the phylum level. Additionally, low-abundance phyla such as Desulfobacterota, Proteobacteria, and Cyanobacteria were detected (Figure 9A). The T2DM group presented reduced abundances of Firmicutes and increased abundances of Bacteroidota relative to the Control group, a finding that is consistent with prior research [24]. Figure 9B shows the distinct abundances of intestinal bacterial genera across the experimental groups. The fecal microbiota of all groups was dominated by *Muribaculaceae*, *Prevotella*, *Romboutsia*, *Eubacterium siraeum* group, *Eubacterium coprostanoligenes* group, *Dubosiella*, *Helicobacter*, *Treponema*, *Prevotellaceae Ga6A1* group, and *Bacteroides*. In the T2DM group, the abundances of *Eubacterium siraeum* group and *Helicobacter* were elevated, while those of *Romboutsia* and *Eubacterium coprostanoligenes* group were reduced. ZJMP intervention upregulated the latter two taxa and downregulated the former two (Figure 9C–F), which indicated that ZJMP restructures the gut microbiota.

Next, LEfSe analysis was used to compare gut microbiota profiles among rat groups. LEfSe results indicated that when the LDA score >3.5 (statistically significant biomarkers distinguishing between groups), 32, 7, 25, 3, 18, and 12 biomarkers were identified in the Control, T2DM, Met, DTH, DTM, and DTL groups, respectively. The LDA score distribution histogram (Figure 9H) showed that key taxa shaping the community structure differed among groups: the Control group was dominated by *g__Romboutsia*, and *g__Eubacterium coprostanoligenes* group; the T2DM group by f__Ruminococcaceae, *g__CAG_352*, *g__Eubacterium siraeum* group, and p__Actinobacteriota; the Met group by *g__Muribaculaceae*, f__Muribaculaceae, and *g__Prevotella*; the DTH group by *g__Parabacteroides*, f__Tannerellaceae, and s__Blautia_faecis; the DTM group by f__Spirochaetota, *g__Treponema*, and o__Spirochaetales; and the DTL group by p__Bacteroidota, o__Bacteroidales, and c__Bacteroidia.

Figure 9I depicts the correlation between ZJMP-modulated key gut microbiota and T2DM-associated indicators. Specifically, *Romboutsia* was strongly positively associated with INS but inversely correlated with HbA1c, FBG, TC, TG, LDH, UA, DAO, and D-LA, which aligns with previous reports of an inverse association between dyslipidemia and *Romboutsia* abundance [25]. The *Eubacterium coprostanoligenes* group exhibited significant negative correlation with FBG, HbA1c, LDL-C, LDH, UA, Cr, IL-6, DAO, and D-LA. The *Eubacterium siraeum* group was significantly positively correlated with multiple indicators, including FBG, HbA1c, LDL-C, TC, TG, CK-MB, LDH, UA, Cr, IL-6, IL-1β, DAO, and D-LA. Additionally, *Helicobacter* was significantly positively correlated with FBG, HbA1c, LDH, UA, Cr, DAO, and D-LA.

### 3.9. ZJMP Regulates Fecal SCFAs Levels in T2DM Rats

**Figure 10:**
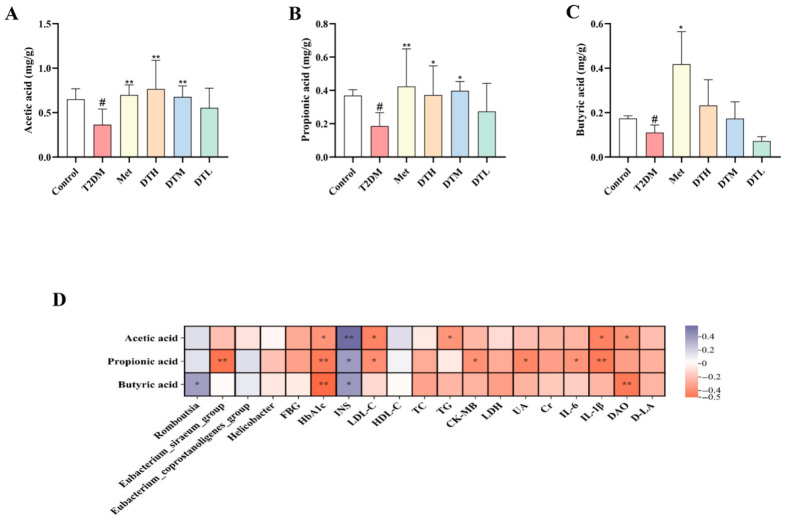
Effects of ZJMP on fecal SCFAs levels in T2DM rats and correlation analysis. (**A**) Acetic acid; (**B**) propionic acid; (**C**) butyric acid; (**D**) correlations among SCFAs, gut microbiota, and biochemical indicators. (*n* = 6). ^#^ *p* < 0.05 vs. Control; * *p* < 0.05, ** *p* < 0.01 vs. T2DM. ZJMP: zaojiaomi polysaccharide; T2DM: type 2 diabetes mellitus; SCFAs: short-chain fatty acids.

The levels of butyric, propionic, and acetic acids were significantly lower in the T2DM group than in the Control group, with this reduction largely restored in the Met group. Additionally, acetic and propionic acid levels were significantly elevated in both the DTH and DTM groups, while butyric acid levels were non-significantly increased. In the DTL group, acetic and propionic acid displayed a non-significant elevation trend, accompanied by a modest reduction in butyric acid (Figure 10A–C).

Correlation analyses identified significant associations of SCFAs with gut microbiota and T2DM-linked biomarkers (Figure 10D). Specifically, acetic acid was negatively correlated with TG, IL-1β, LDL-C, HbA1c, and DAO; propionic acid with HbA1c, IL-1β, LDL-C, IL-6, CK-MB, and UA; and butyric acid with HbA1c and DAO. All three SCFAs were significantly positively correlated with INS. Additionally, propionic acid was negatively correlated with *Eubacterium siraeum* group, which is consistent with our findings. The T2DM group exhibited elevated abundance of this genus and decreased propionic acid levels; in contrast, ZJMP treatment attenuated the genus abundance and significantly increased propionic acid production, with trends opposing those in the T2DM group. Butyric acid was positively correlated with *Romboutsia*, consistent with previous findings that this genus is a butyrate-producing bacterium [26], both butyric acid levels and *Romboutsia* abundance were reduced in the T2DM group; accordingly, they tended to increase following ZJMP intervention. However, butyric acid levels did not increase to a statistically significant extent, suggesting that ZJMP may regulate SCFA production primarily by modulating the *Eubacterium siraeum* group.

## 4. Discussion

T2DM and its complications impose a substantial global health burden, necessitating immediate intervention. In recent years, natural products have exhibited increasingly evident beneficial therapeutic effects in managing T2DM and its complications [27,28]. However, the efficacy and underlying mechanisms of ZJMP against T2DM and its associated cardiac and renal injuries have not yet been fully elucidated. Therefore, we investigated the ameliorative effects of ZJMP on T2DM as well as T2DM-induced cardiac and renal injuries.

Hyperglycaemia is a hallmark feature of T2DM. Prolonged hyperglycaemia impairs insulin receptor function, leading to islet dysfunction, reduced insulin synthesis and secretion, elevated blood glucose and HbA1c levels, and ultimately pathological damage to multiple tissues and organs [29]. In our study, FBG and HbA1c levels were significantly higher in T2DM rats, whereas INS level was markedly lower. Intervention with ZJMP significantly reversed this pathological trend. Additionally, impairment of glucose and lipid metabolism represents a major pathological feature of diabetes mellitus, often leading to insulin resistance, hepatic steatosis, and hyperlipidemia [30]. For T2DM-related dyslipidemia, the primary manifestations include reduced HDL-C concentrations along with elevated LDL-C, TG, and TC levels [31]. Our findings indicate that ZJMP significantly elevated HDL-C levels and reduced LDL-C, TG, and TC levels, thereby alleviating dyslipidemia in T2DM rats. Notably, a non-linear dose–response pattern was observed in the regulation of HDL-C: low and medium doses of ZJMP significantly upregulated HDL-C, whereas the high-dose group only showed a slight upward trend with no statistical significance. This phenomenon has also been documented for some natural plant polysaccharides. A potential explanation is that overly high doses may trigger subtle metabolic feedback regulation or mild intestinal microenvironmental stress, thereby weakening the HDL-C-upregulating effect. Nevertheless, the upward trend of HDL-C in the high-dose group still indicates a potential beneficial lipid-modulating effect.

Studies have confirmed that intestinal mucosal barrier dysfunction is tightly associated with T2DM [32,33]. D-LA is an intestinal bacterial metabolite, and DAO is mainly localized in small intestinal mucosal epithelial cells. Under normal physiological conditions, the intact intestinal barrier prevents the translocation of DAO and D-LA into the bloodstream, resulting in extremely low levels of these two markers in serum [34]. However, metabolic disorders or other factors can induce intestinal flora imbalance and intestinal mucosal barrier damage, which increase intestinal permeability, trigger the massive release of DAO and D-LA into the systemic circulation, thereby elevating their serum levels. We observed that ZJMP significantly reduced DAO levels, while D-LA exhibited a downward trend without reaching statistical significance. This indicates that ZJMP exerted a partial ameliorative effect on intestinal mucosal permeability.

T2DM is frequently accompanied by multiple complications, which tend to damage key organs including the heart and kidneys. Studies have indicated that individuals with T2DM have an 80% risk of cardiovascular or renal complications [35]. Therefore, mitigating diabetes-related complications is pivotal for improving the disease prognosis [36]. CK-MB is predominantly expressed in myocardial cells, and elevated levels of this enzyme are indicative of myocardial injury [37]. In a hyperglycemic environment, increased LDH release reflects enhanced vulnerability of myocardial cells in diabetic patients [38]. UA and Cr are common biomarkers for evaluating renal dysfunction [39]. As anticipated, the biochemical indicators related to cardiac and renal function were significantly elevated in T2DM rats. Following intervention with ZJMP, the levels of these indicators were markedly reduced. Notably, creatinine showed a significant reduction only in the low-dose ZJMP group, while the medium- and high-dose groups merely presented a slight non-significant downward trend. This inconsistent outcome may be explained by a non-linear dose–response effect of ZJMP and individual biological variation among experimental animals. Importantly, the obvious amelioration of renal histological lesions still provides reliable morphological evidence to support the renoprotective capacity of ZJMP. Histological examination revealed varying degrees of pathological damage in the cardiac and renal tissues, whereas ZJMP treatment alleviated pathological lesions in these tissues. Moreover, ZJMP intervention also alleviated pancreatic tissue injury.

In T2DM, elevated IL-1β levels contribute to reduced insulin sensitivity, whereas increased IL-6 levels exacerbate inflammatory responses, aggravate insulin resistance, and impair pancreatic β-cell function [40]. Meanwhile, elevated IL-1β and IL-6 levels are well-recognized risk factors for T2DM-associated complication progression [41]. Evidence indicates that renal injury in T2DM is accompanied by marked upregulation of IL-1β and IL-6 [42,43]. We therefore investigated their expression levels in renal tissues. Our findings confirmed that ZJMP effectively downregulated the increased expression of IL-1β and IL-6 in renal tissues.

Chronic inflammation is a fundamental mechanism underlying T2DM progression and its comorbidities [44]. Among these, TLR4 plays a pivotal role in innate immunity, and its activation initiates downstream signaling cascades to promote the secretion of pro-inflammatory cytokines [45]. TLR4 interacts with its downstream adaptor MyD88 to trigger intracellular signaling cascades, activate the transcription factor NF-κB, and thereby constitute the TLR4/MyD88/NF-κB pathway to mediate inflammatory responses [46]. Targeted modulation of this pathway can significantly alleviate T2DM symptoms and mitigate damage to associated target organs [47,48,49]. To determine whether this pathway is implicated in T2DM-induced renal damage, we assessed the protein expression of TLR4, MyD88, and NF-κB p65. Our results demonstrated a marked reduction in the protein expression of TLR4, MyD88, and NF-κB p65 following ZJMP treatment, which collectively suggests that ZJMP alleviates T2DM-induced renal tissue inflammation by inhibiting the TLR4/MyD88/NF-κB pathway.

In addition to the TLR4/MyD88/NF-κB signaling cascade, the TNFR1 and TNFR2 also play a central regulatory role in the pathogenesis of T2DM and its renal complications [50]. Accumulated evidence demonstrates that activation of TNFR1, and under certain pathological conditions TNFR2, can upregulate IL-1β and IL-6 expression through NF-κB signaling pathways [51,52]. Although we were unable to directly detect the activation status of TNFR1 and TNFR2, our present results showed that ZJMP markedly downregulated the downstream inflammatory mediators IL-1β and IL-6. These findings indirectly suggest that ZJMP may effectively suppress the excessive activation of the TNFR1/TNFR2 inflammatory cascade.

Studies have linked T2DM to gut microbiota dysbiosis. Notably, gut microbiota dysbiosis perturbs intestinal microecological homeostasis and contributes to the development of T2DM via multiple mechanisms, including inducing inflammatory responses, exacerbating insulin resistance, and regulating the production and metabolism of key metabolites such as SCFAs [53]. Therefore, we further investigated the effects of ZJMP on the gut microbiota. Analyses of α and β diversity revealed differences in gut microbiota structures among the groups. Community composition analysis revealed that T2DM decreased the relative abundance of *Eubacterium coprostanoligenes* group and *Romboutsia*, but increased that of *Eubacterium siraeum* group and *Helicobacter*. After intervention with ZJMP, this trend was reversed. Existing studies have shown that *Romboutsia*, a functional bacterial genus associated with obesity, can utilize glucose to produce acetic acid and isobutyric acid, thereby exerting a lipid-lowering effect [54,55]. According to previous studies, the *Eubacterium coprostanoligenes* group, which has been reported as a hub genus in the HFD-induced fecal microecosystem, can mediate HFD-induced dyslipidemia via sphingosine [56]. The *Eubacterium siraeum* group contributes to metabolic disorders by inducing intestinal barrier dysfunction and promoting intestinal inflammatory responses [57]. Additionally, previous research has reported a positive correlation between IL-1β and the *Eubacterium siraeum* group; this suggests that the *Eubacterium siraeum* group may be a core pro-inflammatory genus contributing to T2DM progression [58]. *Helicobacter* is capable of colonizing the stomach, intestines, and liver. It is recognized as a pathogenic gastrointestinal bacterium harmful to human health [59,60]. The aforementioned findings indicate that ZJMP can improve intestinal microecological imbalance by enriching beneficial bacteria such as *Romboutsia* and the *Eubacterium coprostanoligenes* group and simultaneously suppressing the proliferation of harmful bacteria including the *Eubacterium siraeum* group and *Helicobacter*.

Analysis results of key microbial communities and T2DM-related indicators indicate that ZJMP may modulate gut microbiota composition by enhancing the abundances of *Romboutsia* and *Eubacterium coprostanoligenes* group and diminishing those of *Eubacterium siraeum* group and *Helicobacter*. This microbial modulation may subsequently maintain glucose homeostasis, ameliorate lipid metabolism disorders, regulate biochemical indicators related to renal and cardiac function, alleviate inflammation, and preserve intestinal mucosal barrier integrity—collectively mediating the therapeutic effects of ZJMP in T2DM.

As key metabolites derived from gut microbiota, SCFAs exert dual roles in alleviating T2DM: they serve as the main energy substrate for colonic epithelial cells and act as signaling molecules to regulate the functions of various organs via multiple pathways, ultimately enhancing insulin sensitivity and glucose metabolism, and thus regulating T2DM pathogenesis [61,62]. Butyrate, propionate, and acetate account for 95% of total SCFAs [63]. Evidence suggests significantly reduced levels of acetate, propionate, and butyrate levels in T2DM patients [64]. Research has indicated that propionate levels exhibit an inverse association with blood glucose concentrations in individuals with T2DM [65]. Once absorbed into the circulation, propionate further regulates glucose metabolism and appetite [66]. Acetate exhibits potential intestinal barrier-protective effects, which may be mediated by maintaining intestinal integrity and homeostasis, as well as modulating pro-inflammatory immune pathways [67]. In addition, acetate can exert beneficial effects on STZ-induced T1DM mice by ameliorating hyperglycemia and preventing weight loss [68]. Furthermore, combined propionate and acetate administration effectively improves insulin sensitivity and reduces inflammation in diabetic mice [69]. In our present study, ZJMP significantly elevated acetic acid and propionic acid concentrations in T2DM rats. Thus, ZJMP enhances the levels of these two crucial metabolites, which are directly involved in regulating host glucose metabolism and insulin sensitivity.

Notably, although butyric acid is crucial for maintaining intestinal barrier homeostasis and gut health, only a modest increase in butyric acid was observed following ZJMP intervention. This distinct shift in SCFA profiles can be reasonably explained by both the structural characteristics of ZJMP and the metabolic preferences of the intestinal microbiota. First, as a galactomannan polysaccharide, ZJMP is preferentially fermented by gut fermentative microflora to produce acetic acid and propionic acid rather than butyric acid. Butyric acid production is highly dependent on the activity and colonization of specific butyrate-producing bacteria (e.g., *Romboutsia*). Second, under the pathological conditions of T2DM, the colonization and metabolic activity of butyrate-producing microbes are suppressed by chronic hyperglycemia and persistent inflammation. Even after ZJMP intervention, the recovery of butyrate synthetic capacity remains sluggish. Nevertheless, an upward trend in butyric acid was observed; this was significantly correlated with the intestinal injury biomarker DAO, indicating its potential role in repairing intestinal mucosal damage and maintaining intestinal barrier integrity.

Further analysis integrating gut microbiota, SCFAs, and T2DM-associated biomarkers indicates that the primary therapeutic mechanism of ZJMP in ameliorating T2DM involves reducing the abundance of pathogenic gut microbiota (e.g., the *Eubacterium siraeum* group) and promoting SCFA production (particularly acetic acid and propionic acid). These effects subsequently mediate a cascade of downstream beneficial outcomes, including enhancing insulin secretion, alleviating dyslipidaemia, improving intestinal mucosal injury, and suppressing inflammation. It should be emphasized that the above correlation analysis only demonstrates associations among microbiota, SCFAs and biomarkers, and does not confirm definitive causality; further mechanistic verification is still required in future studies. Moreover, while our study focused on SCFAs as representative metabolites, future work could employ comprehensive metabolomic profiling to directly measure a broader spectrum of metabolites and establish their causal links to T2DM amelioration.

## 5. Conclusions

Our findings collectively demonstrate that ZJMP mitigates T2DM and associated cardiorenal injuries through multi-faceted mechanisms involving lipid metabolism improvement, gut microbiota modulation, SCFAs enhancement, and inhibition of the TLR4/MyD88/NF-κB pathway. This work lends strong support to the development of ZJMP as a promising dietary supplement or functional food ingredient for comprehensive T2DM management.

## Data Availability

Data is contained within the article or Supplementary Material.

## Supplementary Materials

The following supporting information can be downloaded at https://www.mdpi.com/article/10.3390/metabo16050339/s1. S1: A chromatographic method for the determination of ZJMP content. S2: A chromatographic method for the determination of SCFA content.

## References

[1] GBD 2021 Diabetes Collaborators (2023). Global, regional, and national burden of diabetes from 1990 to 2021, with projections of prevalence to 2050: A systematic analysis for the Global Burden of Disease Study 2021. Lancet.

[2] Ağagündüz D., Çelik E., Cemali Ö., Yesildemir O., Demirci K.Ş., Akkus G., Esatbeyoglu T., Ozogul F. (2025). Mechanism of actions of probiotics on type 2 diabetes: Development and complications. Biomed. Pharmacother..

[3] Zhao Z., Wu W., Zhang Q., Xing T., Bai Y., Li S., Zhang D., Che H., Guo X. (2025). Mechanism and therapeutic potential of hippo signaling pathway in type 2 diabetes and its complications. Biomed. Pharmacother..

[4] Bailey C.J. (2026). Pharmacological therapies for type 2 diabetes: Future approaches. Diabetologia.

[5] Zhu J., Pan Z., Li D. (2025). Intracellular calcium channels: Potential targets for type 2 diabetes mellitus?. World J. Diabetes.

[6] Sharma P., Sharma S., Ramakrishna G., Srivastava H., Gaikwad K. (2022). A comprehensive review on leguminous galactomannans: Structural analysis, functional properties, biosynthesis process and industrial applications. Crit. Rev. Food Sci. Nutr..

[7] E Y., Chang Z., Lu J., Ju Y., Jiang J., Duan W., Li P., Lei F., Yao X., Wang K. (2023). Enzymatically mediated Gleditsia sinensis galactomannan based hydrogel inspired by wound healing process. Int. J. Biol. Macromol..

[8] Jian H., Cristhian C., Zhang W., Jiang J. (2011). Influence of dehulling pretreatment on physicochemical properties of Gleditsia sinensis Lam. gum. Food Hydrocoll..

[9] Jiang J., Jian H., Cristhian C., Zhang W., Sun R. (2011). Structural and thermal characterization of galactomannans from genus Gleditsia seeds as potential food gum substitutes. J. Sci. Food Agric..

[10] Alsuliam S.M., Albadr N.A., Almaiman S.A., Al-Khalifah A.S., Alkhaldy N.S., Alshammari G.M. (2022). Fenugreek Seed Galactomannan Aqueous and Extract Protects against Diabetic Nephropathy and Liver Damage by Targeting NF-κB and Keap1/Nrf2 Axis. Toxics.

[11] Sun M., Sun Y., Li Y., Liu Y., Liang J., Zhang Z. (2018). Physical properties and antidiabetic potential of a novel galactomannan from seeds of *Gleditsia japonica* var. delavayi. J. Funct. Foods.

[12] Zhu B., Zayed M.Z., Zhu H., Zhao J., Li S. (2019). Functional polysaccharides of carob fruit: A review. Chin. Med..

[13] Feng C., Cheng X., Na M., Zhang F., Duan J., Ji L., Jiang J. (2025). Green preparation of low-molecular-weight galactomannan from Gleditsia sinensis and mechanistic investigation on ameliorating nonalcoholic fatty liver disease. Food Res. Int..

[14] Sang J., Zhao G., Koidis A., Wei X., Huang W., Guo Z., Wu S., Huang R., Lei H. (2024). Isolation, structural, biological activity and application of Gleditsia species seeds galactomannans. Carbohydr. Polym..

[15] Yu X., Li M., Li H. (2025). The role of gut dysbiosis and mitochondrial dysfunction in type 2 diabetes: Insights on pathogenesis, intervention and future perspective. Biomed. Pharmacother..

[16] Liu Y., Li K., Xu J., Shen W., Li Y., Ma J., Wang T., Liu J., Li T., Zhang X. (2025). Alpha-linolenic acid ameliorates T2DM via reshaping gut-liver axis and inflammatory GPR120-NF-κB/NLRP3 pathway in mouse and rat models. Phytomed. Int. J. Phytother. Phytopharm..

[17] Ge X., Du X., Wang Y., Yang Y., Gao X., Zhou Y., Jiang Y., Xiao S., Chen L., Shao R. (2026). Supernatants from Water Extraction-Ethanol Precipitation of Fagopyrum tararicum Seeds Enhance T2DM Management in Mice by Regulating Intestinal Microbial Communities. Foods.

[18] Huang Z., Liu Y., Liu X., Chen K., Xiong W., Qiu Y., He X., Liu B., Zeng F. (2022). Sanghuangporus vaninii mixture ameliorated type 2 diabetes mellitus and altered intestinal microbiota in mice. Food Funct..

[19] Zang Y., Du C., Ru X., Cao Y., Zuo F. (2023). Anti-diabetic effect of modified ‘Guanximiyou’ pummelo peel pectin on type 2 diabetic mice via gut microbiota. Int. J. Biol. Macromol..

[20] Feng Q., Yu X., Xie J., Liu F., Zhang X., Li S., Wang Y., Pan S., Liu D., Liu Z. (2025). Phillygenin improves diabetic nephropathy by inhibiting inflammation and apoptosis via regulating TLR4/MyD88/NF-κB and PI3K/AKT/GSK3β signaling pathways. Phytomed. Int. J. Phytother. Phytopharm..

[21] Guo X., Su Y., Du Y., Zhang F., Yu W., Ren W., Li S., Kuang H., Wu L. (2025). Vinegar-processed Schisandra chinensis polysaccharide ameliorates type 2 diabetes via modulation serum metabolic profiles, gut microbiota, and fecal SCFAs. Int. J. Biol. Macromol..

[22] Li Q., Cheng J., Sun Y., He L., Li R. (2025). Protective Effects of Polygonatum sibiricum Polysaccharides Against Type 2 Diabetic Mice Induced by High-Fat Diet and Low-Dose Streptozotocin. Toxics.

[23] Zhou N., Zhao Y., Zhang L., Ning Y. (2022). Protective effects of black onion polysaccharide on liver and kidney injury in T2DM rats through the synergistic impact of hypolipidemic and antioxidant abilities. Int. J. Biol. Macromol..

[24] Liu J., Wang X., Li Q., Piao C., Fan Z., Zhang Y., Yang S., Wu X. (2023). Fecal metabolomics combined with 16S rRNA gene sequencing to analyze the effect of Jiaotai pill intervention in type 2 diabetes mellitus rats. Front. Nutr..

[25] Chen X., Cai K., Zhang W., Su S., Zhao L., Qiu L., Duan J. (2023). Bear bile powder ameliorates type 2 diabetes via modulation of metabolic profiles, gut microbiota, and metabolites. Front. Pharmacol..

[26] Yu H., Xie Y., Wu B., Zhao H., Chen X., Tian G., Liu G., Cai J., Jia G. (2022). Dietary supplementation of ferrous glycinate improves intestinal barrier function by modulating microbiota composition in Cherry Valley ducks. Anim. Nutr. (Zhongguo Xu Mu Shou Yi Xue Hui).

[27] Niazpour F., Meshkani R. (2025). Natural Products Targeting Ferroptosis in Type 2 Diabetes Mellitus and Its Complications. Phytother. Res..

[28] Liu Z., Wang H., Gong X., Jiang X., Yang K., Jiang L., Wang Z., Tong Q. (2025). Recent advances in natural polysaccharides for type 2 diabetes management: Sources, structural characteristics, and mechanistic insights. Front. Pharmacol..

[29] Cao H., Li C., Lei L., Wang X., Liu S., Liu Q., Huan Y., Sun S., Shen Z. (2020). Stachyose Improves the Effects of Berberine on Glucose Metabolism by Regulating Intestinal Microbiota and Short-Chain Fatty Acids in Spontaneous Type 2 Diabetic KKAy Mice. Front. Pharmacol..

[30] Jiang Y., Gao R., Ying Q., Li X., Dai Y., Song A., Liu H., Hasegawa T., Li M. (2024). Eldecalcitol ameliorates diabetic osteoporosis and glucolipid metabolic disorder by promoting Treg cell differentiation through SOCE. Cell. Mol. Life Sci..

[31] Chen L., Jiang Q., Lu H., Jiang C., Hu W., Yu S., Xiang X., Tan C.P., Feng Y., Zhang J. (2022). Antidiabetic effect of sciadonic acid on type 2 diabetic mice through activating the PI3K-AKT signaling pathway and altering intestinal flora. Front. Nutr..

[32] Qi Y., Li J., Tang Y., Cao R., Gao Y., Xu Q., Han Y. (2024). Total Alkaloids of Rhizoma Corydalis regulates gut microbiota and restores gut immune barrier to ameliorate cognitive dysfunction in diabetic rats. Front. Microbiol..

[33] He Y., Qu C., Zhao H., Wang P., Zheng Z., Miao J. (2024). Conjugated linoleic acid from Suaeda salsa improves the intestinal health in T2DM mice by regulating colonic barrier function, intestinal glycolipid transporters and intestinal flora. Food Biosci..

[34] Peng X., Wei Y., Gong D., Zhang G. (2025). Modulatory Role of Hesperetin-Copper(II) on Gut Microbiota in Type 2 Diabetes Mellitus Mice. Foods.

[35] Colagiuri S., Ceriello A., IDF Technical Working Group (2025). 6. Cardio-renal protection in type 2 diabetes. Diabetes Res. Clin. Pract..

[36] Demir S., Nawroth P.P., Herzig S., Ekim Üstünel B. (2021). Emerging Targets in Type 2 Diabetes and Diabetic Complications. Adv. Sci..

[37] Xiao X., Erukainure O.L., Guo Y., Msomi N.Z., Chu M., Islam M.S. (2025). Jasmine green tea improves glucose homeostasis and antioxidant activities with concomitant hypolipidemic activity in type 2 diabetic rats. Food Biosci..

[38] Yang R., Jia Q., Liu X., Ma S. (2018). Effect of genistein on myocardial fibrosis in diabetic rats and its mechanism. Mol. Med. Rep..

[39] Arigela C.S., Nelli G., Gan S.H., Sirajudeen K.N.S., Krishnan K., Abdul Rahman N., Pasupuleti V.R. (2021). Bitter Gourd Honey Ameliorates Hepatic and Renal Diabetic Complications on Type 2 Diabetes Rat Models by Antioxidant, Anti-Inflammatory, and Anti-Apoptotic Mechanisms. Foods.

[40] Feng Y., Shang B., Yang Y., Zhang D., Liu C., Qin Z., Zhou Y., Meng J., Liu X. (2025). Impact of DPP-4 Inhibitors on Interleukin Levels in Type 2 Diabetes Mellitus. J. Clin. Endocrinol. Metab..

[41] He J., Li X., Yan M., Chen X., Sun C., Tan J., Song Y., Xu H., Wu L., Yang Z. (2024). Inulin Reduces Kidney Damage in Type 2 Diabetic Mice by Decreasing Inflammation and Serum Metabolomics. J. Diabetes Res..

[42] Al-Harbi L.N., Alshammari G.M., Shamlan G., Binobead M.A., AlSedairy S.A., Al-Nouri D.M., Arzoo S., Yahya M.A. (2024). Nephroprotective and Anti-Diabetic Potential of *Beta vulgaris* L. Root (Beetroot) Methanolic Extract in a Rat Model of Type 2 Diabetes Mellitus. Medicina.

[43] Liu D., Chen C., Wang D., Chen Z., Song C. (2020). Effect of sericin on the p38MAPK signaling pathway and NLRP3 inflammasome in the kidney of type 2 diabetic rats. Exp. Ther. Med..

[44] Giardinelli S., Meliota G., Mentino D., D’Amato G., Faienza M.F. (2024). Molecular Basis of Cardiomyopathies in Type 2 Diabetes. Int. J. Mol. Sci..

[45] Kim H., Kim H., Lee J., Hwangbo C. (2023). Toll-like receptor 4 (TLR4): New insight immune and aging. Immun. Ageing.

[46] Coutinho-Wolino K.S., Almeida P.P., Mafra D., Stockler-Pinto M.B. (2022). Bioactive compounds modulating Toll-like 4 receptor (TLR4)-mediated inflammation: Pathways involved and future perspectives. Nutr. Res..

[47] Chen X., Wu J., Fu X., Wang P., Chen C. (2023). Fructus mori polysaccharide alleviates diabetic symptoms by regulating intestinal microbiota and intestinal barrier against TLR4/NF-κB pathway. Int. J. Biol. Macromol..

[48] Liu Y., Tian Y., Dai X., Liu T., Zhang Y., Wang S., Shi H., Yin J., Xu T., Zhu R. (2023). Lycopene ameliorates islet function and down-regulates the TLR4/MyD88/NF-κB pathway in diabetic mice and Min6 cells. Food Funct..

[49] Quan Y., Su P., Shangguan C., Hao H., Yue L., Chen C. (2023). Bergenin ameliorates diabetic nephropathy in C57BL/6J mice by TLR4/MyD88/NF-κB signalling pathway regulation. Toxicol. Appl. Pharmacol..

[50] Su J., Su J., Wang W., Pan J., Zhang X. (2026). Low HDL cholesterol is associated with elevated TNFR1 and TNFR2 levels in early diabetic kidney disease. Front. Endocrinol..

[51] Hayden M.S., Ghosh S. (2014). Regulation of NF-κB by TNF family cytokines. Semin. Immunol..

[52] Siegmund D., Ehrenschwender M., Wajant H. (2018). TNFR2 unlocks a RIPK1 kinase activity-dependent mode of proinflammatory TNFR1 signaling. Cell Death Dis..

[53] Hamamah S., Iatcu O.C., Covasa M. (2024). Nutrition at the Intersection between Gut Microbiota Eubiosis and Effective Management of Type 2 Diabetes. Nutrients.

[54] Fu J., Xiao J., Tu S., Sheng Q., Yi G., Wang J., Sheng O. (2022). Plantain flour: A potential anti-obesity ingredient for intestinal flora regulation and improved hormone secretion. Front. Sustain. Food Syst..

[55] Yang S., Cao S., Li C., Zhang J., Liu C., Qiu F., Kang N. (2022). Berberrubine, a Main Metabolite of Berberine, Alleviates Non-Alcoholic Fatty Liver Disease via Modulating Glucose and Lipid Metabolism and Restoring Gut Microbiota. Front. Pharmacol..

[56] Wei W., Jiang W., Tian Z., Wu H., Ning H., Yan G., Zhang Z., Li Z., Dong F., Sun Y. (2021). Fecal g. Streptococcus and g. Eubacterium_coprostanoligenes_group combined with sphingosine to modulate the serum dyslipidemia in high-fat diet mice. Clin. Nutr..

[57] Yang F., Chen H., Gao Y., An N., Li X., Pan X., Yang X., Tian L., Sun J., Xiong X. (2020). Gut microbiota-derived short-chain fatty acids and hypertension: Mechanism and treatment. Biomed. Pharmacother..

[58] Wu Y., Wang Y., Hu A., Shu X., Huang W., Liu J., Wang B., Zhang R., Yue M., Yang C. (2022). Lactobacillus plantarum-derived postbiotics prevent Salmonella-induced neurological dysfunctions by modulating gut-brain axis in mice. Front. Nutr..

[59] Gorlé N., Bauwens E., Haesebrouck F., Smet A., Vandenbroucke R.E. (2021). Helicobacter and the Potential Role in Neurological Disorders: There Is More Than Helicobacter pylori. Front. Immunol..

[60] Tian J., Zhao X., Tang C., Wang X., Zhang X., Xiao L., Li W. (2023). Protective effect of Paecilomyces cicadae TJJ11213 exopolysaccharide on intestinal mucosa and regulation of gut microbiota in immunosuppressed mice. Food Res. Int..

[61] Liu T., Zhao M., Zhang Y., Xu R., Fu Z., Jin T., Song J., Huang Y., Wang M., Zhao C. (2024). Polysaccharides from Phellinus linteus attenuate type 2 diabetes mellitus in rats via modulation of gut microbiota and bile acid metabolism. Int. J. Biol. Macromol..

[62] Xie C., Qi C., Zhang J., Wang W., Meng X., Aikepaer A., Lin Y., Su C., Liu Y., Feng X. (2025). When short-chain fatty acids meet type 2 diabetes mellitus: Revealing mechanisms, envisioning therapies. Biochem. Pharmacol..

[63] He J., Zhang P., Shen L., Niu L., Tan Y., Chen L., Zhao Y., Bai L., Hao X., Li X. (2020). Short-Chain Fatty Acids and Their Association with Signalling Pathways in Inflammation, Glucose and Lipid Metabolism. Int. J. Mol. Sci..

[64] Salamone D., Costabile G., Corrado A., Della Pepa G., Vitale M., Giacco R., Luongo D., Testa R., Rivellese A.A., Annuzzi G. (2022). Circulating short-chain fatty acids in type 2 diabetic patients and overweight/obese individuals. Acta Diabetol..

[65] Zhang S., Zhang Y., Li J., Wang X., Zhang M., Du M., Jiang W., Li C. (2024). Butyrate and Propionate are Negatively Correlated with Obesity and Glucose Levels in Patients with Type 2 Diabetes and Obesity. Diabetes Metab. Syndr. Obes. Targets Ther..

[66] Shin Y., Han S., Kwon J., Ju S., Choi T.G., Kang I., Kim S.S. (2023). Roles of Short-Chain Fatty Acids in Inflammatory Bowel Disease. Nutrients.

[67] Hernández M.A.G., Canfora E.E., Jocken J.W.E., Blaak E.E. (2019). The Short-Chain Fatty Acid Acetate in Body Weight Control and Insulin Sensitivity. Nutrients.

[68] Zhang C., Wang Z., Luo L., Liu X., Jia Z., Zhang Y. (2025). Acetate Administration Ameliorates Streptozotocin-Induced Hyperglycemia and Adipose Tissue Loss. Faseb J. Off. Publ. Fed. Am. Soc. Exp. Biol..

[69] Mandaliya D.K., Patel S., Seshadri S. (2021). The Combinatorial Effect of Acetate and Propionate on High-Fat Diet Induced Diabetic Inflammation or Metaflammation and T Cell Polarization. Inflammation.

