# Supplementary material for: Polysaccharide from Gleditsia sinensis Seed Endosperm Ameliorates Type 2 Diabetes and Its Associated Cardiorenal Injuries by Modulating TLR4/MyD88/NF-κB Pathway and Gut Microbiota
Source: Metabolites. 2026 May 18;16(5):339. doi: 10.3390/metabo16050339 (PMC13208391; doi:10.3390/metabo16050339)
Supplement: Supplementary file 1 [file metabolites-16-00339-s001.zip › metabolites-4300382-supplementary.pdf]

Supplementary Material

1 A chromatographic method for the determination of ZJMP content

Using pre-column PMP derivatization coupled with high-performance liquid chromatography (HPLC), the monosaccharide composition of ZJMP was analyzed on a Waters 2695 system equipped with a Diamonsil C18 column (5 μm, 250 × 4.6 mm), with acetonitrile (A) and 0.05 M ammonium acetate solution (B) as the mobile phase. The following chromatographic conditions were used: 0–2 min, 18% A; 2–12 min, 18%–20% A; 12–32 min, 20%–22% A; 32–35 min, 22% A; 35–42 min, 22%–40% A. Additional chromatographic conditions were set as: detection wavelength 254 nm, column temperature 40 °C, injection volume 10 μL, and flow rate 1.0 mL/min.

2 A chromatographic method for the determination of SCFAs content

The HPLC analysis was performed on a Waters 2695 system (USA) coupled with a Diamonsil C18 column (5 μm, 250 × 4.6 mm). The mobile phase consisted of acetonitrile (A) and 0.025% phosphoric acid aqueous solution (B), with a gradient elution program as follows: 0–6 min, 2% A; 6–15 min, 2%–4% A; 15–25 min, 4%–10% A; 25–35 min, 10%–20% A; 35–37 min, 20%–26% A; 37–39 min, 26% A (isocratic elution); 39–47 min, 26%–27% A. Additional chromatographic conditions were set as: flow rate 0.8 mL/min, injection volume 10 μL, column temperature 35 °C, and detection wavelength 210 nm.
